# Genome-wide quantification of copy-number aberration impact on gene expression in ovarian high-grade serous carcinoma

**DOI:** 10.1186/s12885-024-11895-6

**Published:** 2024-02-05

**Authors:** Sanaz Jamalzadeh, Jun Dai, Kari Lavikka, Yilin Li, Jing Jiang, Kaisa Huhtinen, Anni Virtanen, Jaana Oikkonen, Sakari Hietanen, Johanna Hynninen, Anna Vähärautio, Antti Häkkinen, Sampsa Hautaniemi

**Affiliations:** 1 Research Program in Systems Oncology, Research Programs Unit, Faculty of Medicine, University of Helsinki, Helsinki, Finland; 2 Institute of Biomedicine and FICAN West Cancer Centre, University of Turku and Turku University Hospital, Turku, Finland; 3 Department of Pathology, University of Helsinki and HUS Diagnostic Center, Helsinki University Hospital, Helsinki, Finland; 4 Department of Obstetrics and Gynecology, University of Turku and Turku University Hospital, Turku, Finland; 5 Foundation for the Finnish Cancer Institute, Helsinki, Finland; 6 Computational Health Informatics Program, Boston Children’s Hospital, Harvard Medical School, Boston, MA USA

**Keywords:** Copy-number alterations, Gene expression, Statistical modeling, Data integration, Pathway analysis, Ovarian cancer, Drug resistance, *KRAS*

## Abstract

Copy-number alterations (CNAs) are a hallmark of cancer and can regulate cancer cell states via altered gene expression values. Herein, we have developed a copy-number impact (CNI) analysis method that quantifies the degree to which a gene expression value is impacted by CNAs and leveraged this analysis at the pathway level. Our results show that a high CNA is not necessarily reflected at the gene expression level, and our method is capable of detecting genes and pathways whose activity is strongly influenced by CNAs. Furthermore, the CNI analysis enables unbiased categorization of CNA categories, such as deletions and amplifications. We identified six CNI-driven pathways associated with poor treatment response in ovarian high-grade serous carcinoma (HGSC), which we found to be the most CNA-driven cancer across 14 cancer types. The key driver in most of these pathways was amplified wild-type *KRAS*, which we validated functionally using CRISPR modulation. Our results suggest that wild-type *KRAS* amplification is a driver of chemotherapy resistance in HGSC and may serve as a potential treatment target.

**Supplementary Information:**

The online version contains supplementary material available at 10.1186/s12885-024-11895-6.

## Background

Copy-number alterations (CNAs) are a hallmark of cancer and play a critical role in cancer progression and therapy resistance by modulating the expression levels of genes [1–3]. Gene activity at the mRNA-level, however, is regulated by several mechanisms in addition to CNAs, such as DNA methylation and micro-RNAs, which make transcriptomics analysis challenging. Thus, it is important to systematically identify genes whose expression values are strongly influenced by CNAs as these genes may have fundamental importance in cancer progression and therapy resistance. Moreover, owing to the superior stability of DNA compared to RNA, copy-number alterations are often favored as biomarkers in routine diagnostics over gene expression assays.

Several computational methods have been developed to identify genes that exhibit concomitant changes in CNAs and expression levels from genomic data [4]. However, none of the existing methods explicitly quantify the degree to which a gene’s expression value is regulated by CNAs or enable unbiased categorization of the CNAs, such as amplifications or deletions. Furthermore, many of these methods suffer from a high rate of false positives [4]. which can compromise the accuracy of downstream analyses and lead to erroneous conclusions.

To identify genes and pathways that are likely regulated by CNAs, we developed a statistical model that estimates gene-specific copy-number impact (CNI) values, quantifying the degree to which CNAs are associated with alterations in gene expression levels. We show that CNI differs from the abundance of CNAs, and the landscape of the two can be used to discriminate between CNAs that potentially drive changes in gene expression and those that do not.

We applied our CNI approach to ovarian high-grade serous carcinoma (HGSC), the most common and lethal subtype of epithelial ovarian cancers with five-year survival of < 40% [5–7]. HGSC is known for high genomic instability and extensive CNAs due to *TP53* mutations and is considered as a copy-number driven cancer [8]. Still, except for a few genes, such as *CCNE1* [9, 10], or *NCOA3*-adjacent [9, 10] amplifications, the specific CNAs that govern therapy response in HGSC remain poorly understood. To identify the genes and pathways that are regulated by CNAs, we utilized whole-genome sequencing (WGS) and RNA-seq data from 263 samples derived from 93 patients with HGSC enrolled in the DECIDER study (Multi-layer Data to Improve Diagnosis, Predict Therapy Resistance and Suggest Targeted Therapies in HGSOC; ClinicalTrials.gov identifier: NCT04846933).

## Methods

### DECIDER cohort patients and samples

The prospective DECIDER cohort consists of patients with HGSC who have undergone primary debulking surgery (PDS) or neoadjuvant chemotherapy (NACT) treated at Turku University Central Hospital. NACT consisted of a median of three (range 3–4) cycles of carboplatin and paclitaxel chemotherapy. The subcohort used in this project consists of 475 bulk RNA-seq and 364 whole-genome sequencing samples (with purity > 5%), of which 263 samples of 93 patients are matching (Supplementary Table 6). There were 39 PDS and 54 NACT patients in the analysis, among which there were 177 treatment-naïve (before chemotherapy), 56 post-treatment (after neoadjuvant chemotherapy), and 30 relapse samples (after recurrence). Tumor tissues include primary tubo-ovarian tumors, other intra-abdominal (omentum, peritoneum, mesenterium), and tumors from other sites (including ascites). All patients participating in the study gave their informed consent, and the study was approved by the Ethics Committee of the Hospital District of Southwest Finland.

### DECIDER whole-genome sequencing preprocessing

Whole-genome sequencing and data preprocessing was performed as described in [11]. The preprocessing steps include quality control and trimming (using FastQC [12] version 0.11.4, Trimmomatic [12, 13] version 0.32), alignment to the reference genome (GRCh38.d1.vd1), duplicate marking (Picard [14] MarkDuplicates version 2.6), base quality score recalibration (GATK [15] BaseRecalibrator [16] version 3.7) and contamination estimation (GATK version 4.1.9.0). All the steps were performed using Anduril 2, a bioinformatics workflow platform developed for large data sets [17].

### Absolute copy-number estimation

We used GATK version 4.1.4.1 to perform the CN segmentation. The analysis pipeline follows the GATK best-practices documentation [18] and builds upon the Anduril 2 platform [17].

To collect the minor allele counts (b-allele frequency, BAF), we used all the filtered biallelic germline SNPs, genotyped using GATK version 4.1.9.0 joint calling [19] with heterozygous calls (VAF between 40 and 60%) from each patient. Read-count collection used one kilobase intervals. Both read and allelic count collection excluded regions listed in the ENCODE blacklist [20]. We used platform specific (HiSeq, DNBSEQ) panels of normals built from the normal samples to denoise the read counts.

To estimate purity, ploidy, and allele specific CNAs, we used a re-implemented ASCAT algorithm [21]. The original ASCAT R package was not directly applicable because it does not accept data segmented using external tools. Our implementation also uses the variant-allele frequency (VAF) of truncal pathogenic *TP53* mutation as additional evidence for the optimal ploidy/purity selection. As nearly all HGSC patients have a homozygous *TP53* mutation in the cancer cells, the VAF can be used to the estimate of the total CN to approximate the purity:

$$\mathrm{purity}\:=\:2/\left(\left(\mathrm{CN}/\mathrm{VAF}\right)-\left(\mathrm{CN}\:-\:2\right)\right).$$

Patients having discordant ploidy estimates between the samples went through manual curation. The CN of each gene is based on the longest segment that intersects the gene.

### DECIDER bulk RNA-seq preprocessing

Sequencing libraries for 475 samples were performed with DNBSEQ, HiSeq X Ten or Illumina Hiseq 4000 (Illumina, USA), as 100 bp or 150 bp paired end sequencing producing around 60 M reads.

The data were then processed using SePIA, a comprehensive RNA-seq data processing workflow [22]. Read pairs were trimmed using Trimmomatic [13] version 0.33 as follows: (i) the first 12 and last 5 bases were cropped due to uneven per base sequence content; (ii) any leading bases with a quality score < 20 and any trailing bases with a quality score < 30 were removed; (iii) the reads were scanned with a 3-base wide sliding window, cutting when the average quality per base dropped < 20; (iv) resulting sequences < 20 bp were discarded. Trimmed reads were aligned to the reference genome (GRCh38.d1.vd1) using STAR [23] version 2.5.2b, allowing up to 10 mismatches, and all alignments for a read were output. Gene level expression was quantified using eXpress [24] version 1.5.1-linux_x86_64.

### Bulk RNA-seq decomposition

The bulk RNA samples were decomposed into cancer, immune, and stromal components using PRISM [25]. The decomposition provides both sample composition and expression profiles for each individual sample and cell type. The decomposition was guided by single-cell data of 16,826 cells from 15 patients (18 samples) from multiple tissue sites that were isolated from partially matching DECIDER patient samples [26]. The cell types were annotated using shared nearest neighbor modularity clustering from Seurat version 2.3.4 [27] based on the following markers: *WFDC2*, *PAX8*, *MUC16*, *EPCAM*, *KRT18* (epithelial); *COL1A2*, *FGFR1*, *DCN* (stroma); *CD14*, *CD79A*, *FCER1G*, *PTPRC*, *NKG7*, *CD3D*, *CD8A* (immune). The cancer cell specific expression was extracted for the current analysis, while the signal from the non-cancerous cells was discarded to match the WGS data.

Batch effects between the samples with different library preparation protocols were corrected using POIBM [28] by mapping all the samples to the space of the larger dataset using the default parameters.

### TCGA copy-number and bulk RNA-seq data

TCGA gene level absolute CNAs and bulk RNA gene expression data of 14 cancer types were downloaded from the Broad Firehose [29] on February 16 2022. The list of the cancer types and the number of patients used in each specific analysis are reported in Supplementary Table 7. Clinical annotations for each data set were also downloaded, and the available overall survival (OS) data were included in our analysis. RNA-seq decomposition was implemented for the TCGA OV cohort using the same single-cell reference set as for the DECIDER HGSC cohort. For SKCM, 4,097 single-cells classified as ‘Melanoma’, ‘B’, ‘T’, ‘Macrophage’, ‘Endothelial’, ‘CAF’, or ‘NK’ in [30] (available from Gene Expression Omnibus under the identifier GSE72056) was used as the reference. For GBM, 3,381 single cells classified as ‘Neoplastic’, ‘Myeloid’, ‘Astrocytes’, ‘Oligodendrocytes’, or ‘OPCs’ in both malignant and non-malignant tissues in [31] (GSE84465) was used as a reference in the PRISM [25] decomposition. In each case, the non-cancerous signal was discarded to match the signal from the CNA data.

### Copy-number expression model

*Linear and nonlinear Poisson models:* Since gene expression values are discrete, non-negative, and possess widely varying noise levels [32] we developed an approach based on a Poisson model to extract the gene expression variation explained by their corresponding CNAs. For each individual gene, we first considered a constant model modeling gene expression in the absence of any CNA induced changes, as X_ij_ ~ Poi( b_i,0_ g_j_) st. b_i,0_ ≥ 0 where X_ij_ denotes the expression of the i:th gene in the j:th sample, g_j_ the global expression level of the j:th sample, and b_i,0_ denotes a constant (basal) expression level for the i:th gene. We also made two linear models, one with no interaction effect to act as a null model for testing the asymmetric relation, as X_ij_ ~ Poi( ( b_i,0_ + a_i_ ( A_ij_ + B_ij_) g_j_) st. b_i,0_, a_i_ ≥ 0, where A_ij_ and B_ij_ are the major and minor absolute CNA values for the i:th gene and the j:th sample, respectively, and a_i_ is the linear CNA effect; and another including an interaction term capturing asymmetries in the major and minor numbers:

$${\mathrm X}_{\mathrm{ij}}\:\sim\:\mathrm{Poi}\left(\left({\mathrm b}_{\mathrm i,0}\:+\:{\mathrm a}_{\mathrm i}\left({\mathrm A}_{\mathrm{ij}}\:+\:{\mathrm B}_{\mathrm{ij}}\right)+{\mathrm c}_{\mathrm i}\;{\mathrm A}_{\mathrm{ij}}\;{\mathrm B}_{\mathrm{ij}}\right){\mathrm g}_{\mathrm j}\right)\;\mathrm{st}.\;{\mathrm b}_{\mathrm i,0},\;{\mathrm a}_{\mathrm i}\:\geq\:0,$$

where c_i_ > 0, c_i_ = 0, c_i_ < 0 denote synergistic, linear, and antagonistic interaction between the major and minor numbers. All models were fit in maximum likelihood sense using PRISM [25]. Afterwards, we tested for any effect of CNAs over gene expression using a likelihood ratio test between the null and the alternative models.

To capture a nonlinear CNA effect on gene expression variation, we applied the following monotonic Poisson model:

$${\mathrm X}_{\mathrm{ij}}\:\sim\:\mathrm{Poi}\left(\left({\mathrm b}_{\mathrm i,0}\:+\:{\mathrm a}_{\mathrm i,1-0}\left(1_{\mathrm{Aij}\;\geq\;1}\:+\:1_{\mathrm{Bij}\;\geq\;1}\right)+\:{\mathrm a}_{\mathrm i,2-1}\left(1_{\mathrm{Aij}\;\geq\;2}\:+\:1_{\mathrm{Bij}\;\geq\;2}\right)+\:\dots\right){\mathrm g}_{\mathrm j}\right)\;\mathrm{st}.\;{\mathrm b}_{\mathrm i,0},\;{\mathrm a}_{\mathrm i,\mathrm k-(\mathrm k-1)}\:\geq\:0,$$

where 1_E_ is an indicator for the expression E, and a_i,k-(k-1)_ represent the major and minor allele specific CNA effects when the CNA increases from k-1 to k in the i:th gene. A non-linear model was found to outperform a linear model significantly (see Supplement).

*Stacked nonlinear response models:* To identify if the CNA impact over gene expression varies systematically over the response groups, we implemented a nonlinear monotonic Poisson model, where the response model has a constant effect direction between the good and poor responder groups. The constant general model was used as the null model. As the alternative model, we used a stacked form where the gene expression is modeled via two models, one for the good responders as

$${\mathrm X}_{\mathrm{ij}}\:\sim\:\mathrm{Poi}\left(\left(\left({\mathrm b}_{\mathrm i,0}\:+\:{\mathrm d}_{\mathrm i,0}\right)+\left({\mathrm a}_{\mathrm i,1-0}\:+\:{\mathrm d}_{\mathrm i,1-0}\right)\left(1_{\mathrm{Aij}\;\geq\;1}\:+\:1_{\mathrm{Bij}\;\geq\;1}\right)+\:\dots\right){\mathrm g}_{\mathrm j}\right)\;\mathrm{st}.\;{\mathrm b}_{\mathrm i,0},\;{\mathrm d}_{\mathrm i,0},\;{\mathrm a}_{\mathrm i,\mathrm k-(\mathrm k-1)},\;{\mathrm d}_{\mathrm i,\mathrm k-(\mathrm k-1)}\:\geq\:0,$$

where d_i,k-(k-1)_ ≥ 0 are the additional good responder specific deviations, and the model for the poor responders is as specified previously. A corresponding model with positive poor responder specific deviations was also fitted, and the better fitting model selected as the final one to quantify the expression differences between the groups.

*Stacked nonlinear phase model.* To characterize if the CNA effect over gene expression changes is different over treatment, we also implemented a nonlinear monotonic Poisson model where the treatment model has a constant effect direction from diagnosis to post treatment group. As with the poor and good responder group models, two submodels were used, one for the diagnosis samples and one for the post treatment samples. Both effect directions were fitted, and in the final model some genes had a positive effect changing from the diagnosis to post-treatment while others showed the positive effect from post-treatment to diagnosis.

*Statistical tests and correlation coefficients:* The generalized correlation of the linear/nonlinear model was calculated as the square root of the explained variance, in which the explained variance is measured as the R-squared values of linear/nonlinear model over the constant model. A Likelihood Ratio Test with test statistics equal to the log-likelihood differences between the constant and the linear/nonlinear model and degree of freedom as the difference between the rank of the linear/nonlinear and the constant one was used to compute a p-value following Wilks’ theorem. For the pathway/gene-set level absolute value of correlation, the R-squared values of the linear/nonlinear model and the constant model were aggregated separately for all the genes of a pathway, and the generalized correlation coefficient for the collection of the models was computed accordingly.

### Pathway enrichment analysis

First we calculated the ssGSEA [33] scores for the 18 pathways enriched in poor responders using all 177 treatment-naïve samples from 88 patients and tested for association with the platinum-free interval (PFI) as described below. In the analysis, each patient was represented by the ssGSEA score from the sample with the highest tumor purity.

### Survival analysis

Kaplan–Meier (K-M) curves were estimated for survival analysis based on either platinum-free interval (PFI) or overall survival (OS). PFI is defined as the time from the last cycle of platinum treatment to cancer progression or to the last follow-up, whereas OS is the time from diagnosis to death or the last follow-up. A log-rank test was used to test the significance of the PFI/OS-based survival differences. To optimize the threshold for grouping the patients in the survival analysis, we used maximally selected rank statistics (max.stat) thresholding method in R [34] which appropriately controls the significance for the threshold optimization.

### Clustering of CNA versus CNI

As the TCGA CN and mutation driver gene sets are naturally reside in the high CNA/high CNI and low CNA/low CNI regions, respectively, we used these genes to optimize clustering for the CNA versus CNI landscape. The pathways were clustered based on the CN and mutation driver pathway distributions such that for the CN pathways, the known mutation driver genes from each individual pathway were replaced with randomly sampled genes selected from the whole genome. The sampling was repeated 1,000 times to mitigate random effects. The same procedure was repeated for the mutation driver pathways, and the process resulted in two distributions of mutation and CN driver specific pathway statistics. Finally, the threshold that optimized the clustering accuracy based on the two distributions in the CNI and the average CNA axes were acquired independently.

### Gene specificity of the pathway aberrations

To assess the specificity of CNI at the pathway level, we quantified the contribution of the involved genes to the aggregate pathway CNI. This was implemented through a leave one out procedure in the nonlinear model, which allowed us to quantify how much impact each gene contributed to the overall correlation coefficient over the pathway. From the relative explained variance by each involved gene, we calculated per-pathway perplexity values representing the effective number of genes contributing to pathway activity for each pathway. The perplexity π is computed as follows:

$$\pi =exp\left(-{\sum }_{i=1}^{k}{p}_{i}.log\left({p}_{i}\right)\right),$$

where *p*_*i*_ is the relative contribution (normalized fraction of explained variance) of the *i*^th^ gene over the *k* involved genes in the pathway, and 0.log(0) = 0.

### Functional transition point of the CNA and its variability

To probe the functional CNA for each gene, we analyzed the fitted expression model curves across all samples. The median and the lower and upper quartiles of the gene expression were projected on the nonlinear model curve, and the corresponding CNAs were interpolated to quantify the transition point and the transition bandwidth of the model curve. Based on the resulting CNAs, the genes were further categorized into five groups as genes with deletion (CNA = 0), loss (CNA = 1), normal (CNA = 2), gain (2 < CNA ≤ 5), and amplification (CNA > 5). The category with the most hits was then chosen to obtain the pathway level transition points and their variability.

### Cell culture

KURAMOCHI cells (JCRB Cell Bank, JCRB0098) were grown in RPMI1640 (Corning, 10–040-CV) with 10% fetal bovine serum (FBS) (Gibco, 10,270,106), 1% penicillin–streptomycin (PS) (Gibco, 15,140,122). CAOV3 cells (ATCC, HTB-75) were grown in DMEM (Corning, 15–013-CV) with 10% FBS, 1% PS, and 1% GlutaMAX (Gibco, 35,050,038).

### Plasmid construction

The Lenti-idCas9-KRAB-neo and Lenti-idCas9-VP64-neo plasmids were generated from Lenti-iCas9-neo (Addgene, plasmid # 85,400), TRE-KRAB-dCas9-IRES-GFP (Addgene, plasmid # 85,556) and lenti dCAS-VP64_Blast (Addgene, plasmid # 61,425). The lentiMPH v2-EGFP plasmid was generated by replacing hygromycin with EGFP from lentiMPH v2 (Addgene, plasmid # 89,308). CROPseq-i-sgRNA-BFP and CROPseq-a-sgRNA-BFP backbones were generated from CROPseq-Guide-Puro (Addgene, plasmid # 86,708). CROPseq-i-NC-BFP and CROPseq-i-KRAS-BFP plasmids were generated from CROPseq-i-sgRNA-BFP backbone with i-NC or i-KRAS, respectively. CROPseq-a-NC-BFP. CROPseq-a-KRAS-1-BFP and CROPseq-a-KRAS-2-BFP plasmids were generated from CROPseq-a-sgRNA-BFP backbone with a-NC, a-KRAS-1, or a-KRAS-2, respectively. The sgRNA sequences are listed in Supplementary Table 8. Lenti-iCas9-neo was a gift from Qin Yan, TRE-KRAB-dCas9-IRES-GFP was a gift from Eric Lander, lenti dCAS-VP64_Blast and lentiMPH v2 were gifts from Feng Zhang, and CROPseq-Guide-Puro was a gift from Christoph Bock [35–39].

### Lentivirus production and cell transduction

Lentiviral particles were produced by transfecting 293FT cells (Thermo Fisher, R70007) with each target plasmid, along with packaging constructs psPAX2 (Addgene, plasmid # 12,260), and pMD2.G (Addgene, plasmid # 12,259). The lentivirus were collected 48 h and 72 h after transfection and aliquoted for storage at -80 °C, following filtering through a low-protein binding 0.45-μm filter. To generate stably transduced cell lines, cells were infected with the lentivirus for 24 h and then either selected for 7 days in the corresponding culture medium with 400 μg/mL G418 (ThermoFisher Scientific, 10,131,035) or FACS sorted for GFP/ BFP positive populations with BD FACSAria II cell sorter.

### Quantitative real-time polymerase chain reaction (qRT-PCR)

RNA was isolated by using NucleoSpin® RNA Plus kit (Macherey–Nagel, 740,984,250), and reverse-transcription was performed with iScript cDNA Synthesis Kit (Bio-Rad, 1,708,891) according to the handling procedure. qRT-PCR was performed with PowerUp™ SYBR™ Green Master Mix (ThermoFisher Scientific, A25776). Three replicates were performed. The primer sequences are listed in Supplementary Table 8.

### Apoptosis assay

One hundred thousand cells were seeded in 6-well plates and treated with individual drugs for 3 days. Media and cells were collected and washed with PBS twice. Cells were resuspended with 100 μl of 1X annexin-binding buffer (ThermoFisher Scientific, V13246), 5 μl of Annexin V-APC (ThermoFisher Scientific, R37176) and 1 μl of 100 μg/ml propidium iodide (PI) (ThermoFisher Scientific, P3566). Cells were incubated at room temperature for 15 min in dark, then added 400 μl of 1X annexin-binding buffer. Quantification was performed with flow cytometry NovoCyte Quanteon Analyser. Three replicates were performed.

### Colony formation assay

Cells were seeded in 6-well plates (2 000 cells for the untreated group, and 10,000 for platinum groups), cultured overnight, treated with platinum for 7 days, and cultured with complete media for another 7 days. Then colonies were washed with PBS once, fixed with methanol for 10 min, and stained with crystal violet for 20 min. Three replicates were performed. The colony intensity quantification is done using the ColonyArea plugin in the ImageJ software [40].

## Results

### Gene level quantification of CNA impacted expression in 14 cancers

**Fig. 1:**
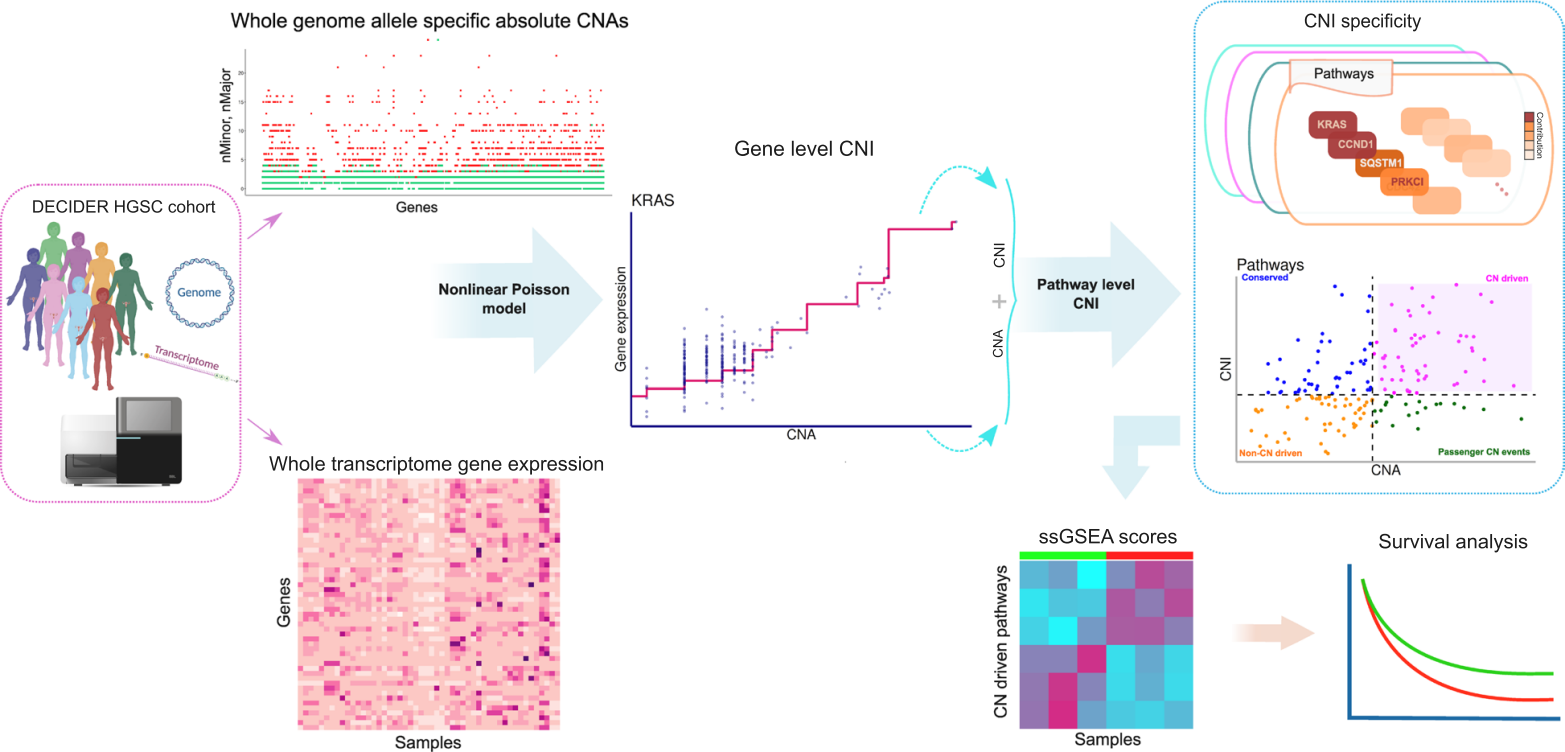
Overview of the material, model and CNI capture Allele specific copy-number values per gene and the gene expression profiles across different samples are used as input in the model. A nonlinear Poisson model is implemented to quantify the CNI over gene expression for each individual gene, and the results are pooled statistically to obtain a pathway level CNI. Afterwards, the CNI and absolute CNAs are used to create a landscape of CNA functionality at pathway level. A thresholding method is applied to cluster the landscape resulting into CN/non-CN aberrant and CN/non-CN-driven pathways simultaneously. The pathway level ssGSEA scores are computed using gene expression of CN-driven pathways enriched in poor and good responding patients. A survival analysis reveals prognostic pathways for all DECIDER cohort patients at diagnosis

To identify genes whose expressions are regulated by CNAs, we developed a nonlinear monotonic Poisson regression model (Fig. 1. Methods, Supplement). The inputs of the model are RNA-seq gene expression and allele specific absolute CNAs estimated by ASCAT [21] for matching samples. The model can be used to extract copy-number impact (CNI) values, which quantify the degree of a gene’s expression variation directly exerted by the CNAs in the same chromosomal region. The CNI values were obtained by taking the square root of the explained variance, defined as the R-squared values of the linear/nonlinear model over the constant model (see Methods). Examples of the model curves with various CNI values for six genes (*RB1*, *ERBB2*, *CCNE1*, *FOXA1*, *MET* and *MUC4*) are shown in Supplementary Fig. 1.

**Fig. 2:**
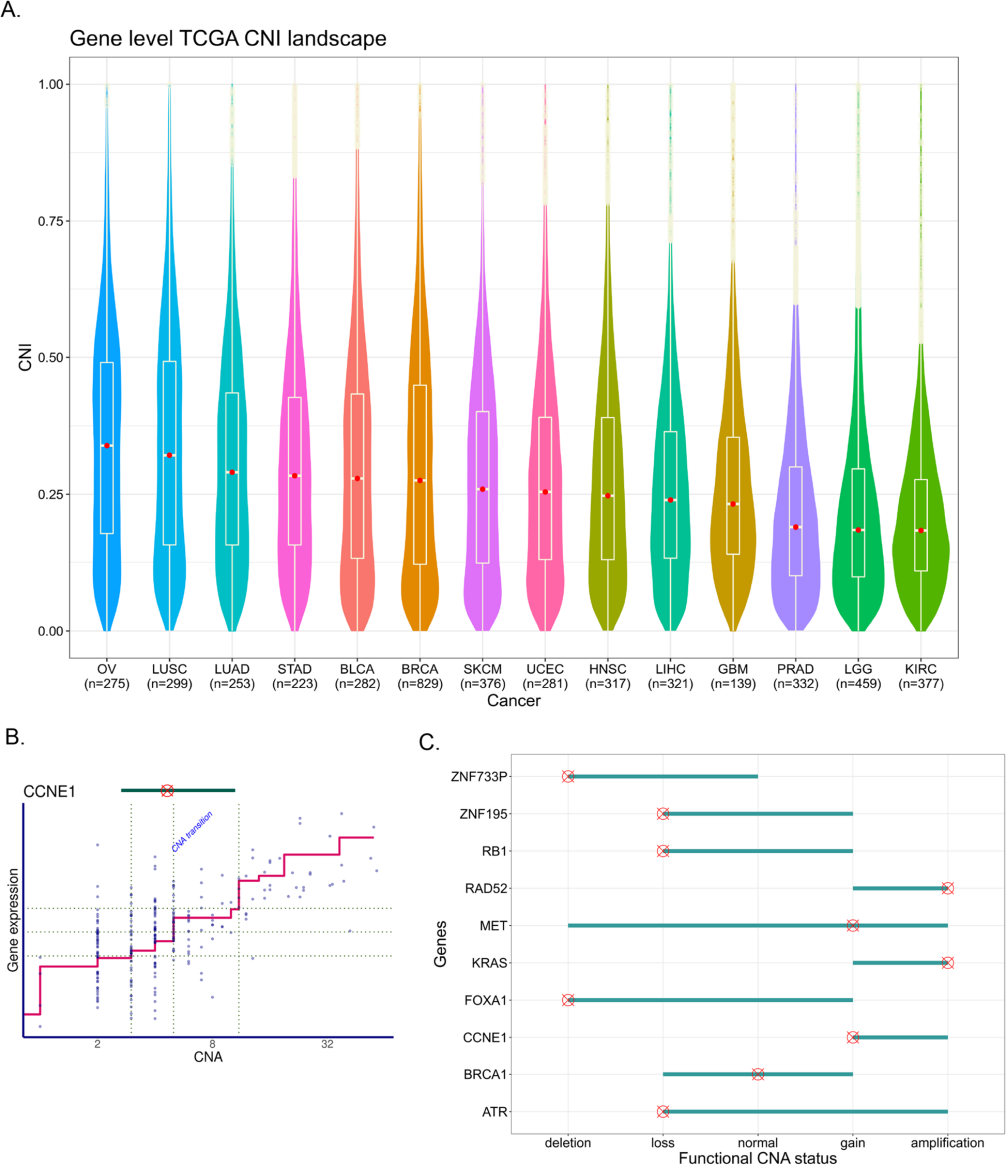
CNI and functional CNA levels at gene level **A**. The spectrum of CNI across 14 TCGA cancer types using raw bulk RNA-seq expression data and absolute CNAs. The model was applied on the TCGA OV, LUSC, LUAD, STAD, BRCA, BLCA, HNSC, UCEC, SKCM, GBM, LIHC, LGG, PRAD, and KIRC datasets, allowing the quantification of CNI in each. The dashed line shows the median CNI. The results indicate that OV lands on the high extreme of the CNI, along with LUSC, LUAD, STAD, and BRCA, while KIRC, GBM, SKCM, and LGG are located in the lower tail of the CNI spectrum. Nine of these 14 cancer types have been shown to have a similar trend in CNA abundance in previous literature, suggesting the abundance correlates with but does not equate CNA driverness. **B**. CNA transition point captured from nonlinear model curve as functional CNA level for *CCNE1* as an example gene. **C**. The obtained functional CNA status (red target) as the point with the highest impact over gene expression and its range of variability (cyan bar) across the DECIDER cohort are visualized for some example genes including *ATR*, *BRCA1*, *CCNE1*, *FOXA1*, *KRAS*, *MET*, *RAD52*, *RB1*, *ZNF195*, and *ZNF733P*

Cancers can be considered as mutation- or CNA-driven [6]. However, as earlier studies have not considered the functional impact of CNAs on expression values in their analyses, we first compared the degree to which CNAs impact gene expression values in 14 different cancer types. Accordingly, we calculated CNI values for each gene across for 14 cancers using The Cancer Genome Atlas (TCGA) data. The CNI distribution and their ranking are provided in Fig. 2A. HGSC exhibit the highest CNI values followed by lung squamous cell carcinoma (LUSC) and lung adenocarcinoma (LUAD), whereas glioblastoma multiforme (GBM) and brain lower grade glioma (LGG) showed low CNI values.

To illustrate further CNI analysis across three cancer types, we selected HGSC, melanoma, which is less driven by CNAs than HGSC [41] and glioblastoma multiforme (GBM), which shows very low CNI and is considered as a mutation-driven cancer in earlier studies [42]. These three cancer types also have single-cell RNA-seq data available that enables decomposition of bulk RNA-seq into cancer cell specific gene expression values. As patient-derived samples have wide variability of tumor cell fraction, analysis without decomposition may lead to confounding results [25]. Herein we used PRISM [25] which uses a latent statistical framework to extract cell-type specific transcriptome profiles. After decomposition, the cancer cell specific gene expression values and whole-genome allele-specific CNAs were used to calculate CNI values. The obtained CNI value distributions over all genes corroborate the known role of CNAs in these cancers, with HGSC having a high number of genes whose expression is driven by CNAs, melanoma, substantially fewer, and glioblastoma multiforme only a very low number (Supplementary Fig. 2). Additionally, our analysis of the established TCGA CN and mutation driver genes [43] in the DECIDER and TCGA cohorts show that the potential CN drivers predominantly exhibit high CNI and CNAs (see Supplementary results and Supplementary Table 1) while potential mutational drivers have low CNI with fewer CNAs.

Identification of categories, *i.e.*, deletions, losses, gains, and amplifications, is important for interpretation of the CNA-based analysis. However, currently the thresholds for these categories are done manually and they may differ substantially between studies for the same gene. The CNI model allows unbiased categorization, which is based on the functional impact a CNA level has to gene expression values. This is based on obtaining the transition point of the model curve*, i.e.,* the CNA that exerts an expression change (Methods). An example of a transition point and bandwidth for the *CCNE1* gene in the DECIDER cohort is shown in Fig. 2B. A low transition point indicates a functional change due to deletions/losses, and a high one due to gains/amplifications. The corresponding transition bandwidth characterizes the range of CNAs that modulate expression (Supplementary Table 2). Examples of the transition points and bandwidths for 10 genes (*ATR*, *BRCA1*, *CCNE1*, *FOXA1*, *KRAS*, *MET*, *RAD52*, *RB1*, *ZNF195*, *ZNF733P*) in DECIDER cohort are shown in Fig. 2C. For instance, *CCNE1* expression changes are due to gains (2 < CNA ≤ 5) and amplifications (CNA > 5) only, whereas for *MET,* any type of CNAs ranging from deletion (CNA = 0) to amplification (CNA > 5) is reflected in the expression level.

### Pathway level analysis with CNI and CNAs identifies enriched pathways in poor and good responding patients with HGSC

**Fig. 3:**
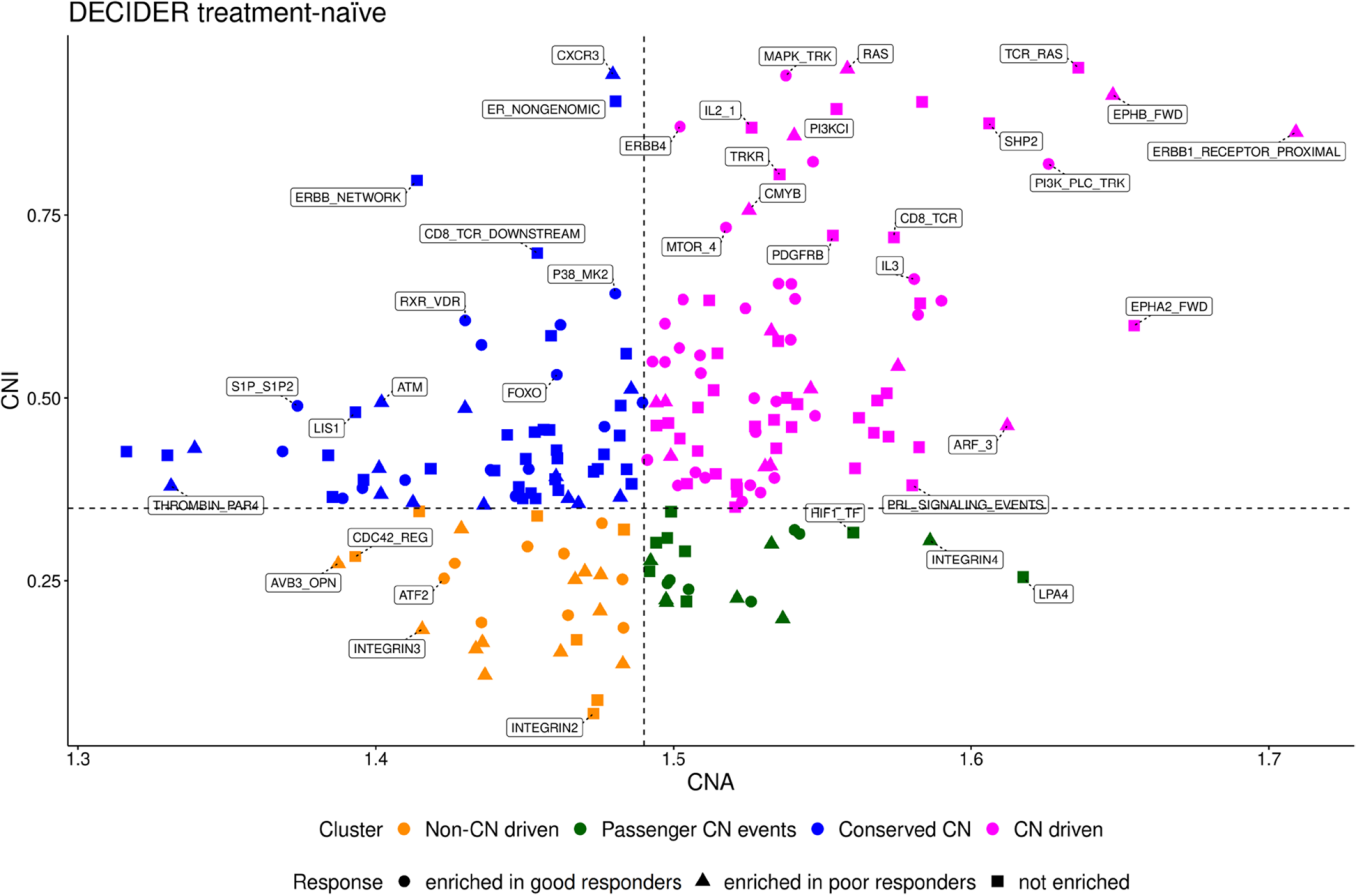
The landscape of CNA versus CNI in the DECIDER treatment-naïve samples CNA versus CNI landscape for the response model based on the general model’s thresholding in the treatment-naïve samples. The magenta points represent the pathways in CN-driven cluster, blue the conserved, orange the non-CN-driven and the green shows pathways with passenger CN events, while those pathways enriched in poor responders, good responders and non-enriched ones are shown via triangles, circles and squares respectively

To test whether high CNI at the pathway level is reflected in patients’ response to therapy at diagnosis, we used a stacked nonlinear version of our model (Methods) and analyzed 133 treatment-naïve samples including 76 samples from patients with good response (platinum-free interval > 12 m) and 57 samples from patients with poor response (platinum-free interval ≤ 6 m) to therapy. Pathway level values were computed from the gene level CNI values among the 196 curated Pathway Interaction Database (PID) pathways [44, 45] (Supplementary Fig. 3). Each pathway was visualized in a space described by the average of absolute CNAs across all samples and CNI as axes, and then assigned to one of the four segments (Methods) as illustrated in Fig. 3.

First, the pathways characterized by both high CNI and high CNAs highlight putative CN-driven pathways. Second, the segment with low CNI and low CNAs contains pathways whose activity is not driven by CN changes, but are controlled by other means. Third, the segment that contains pathways with high CNI and low CNAs are named as conserved CN pathways, because these pathways are highly impacted by CNAs but only minor CNAs manifest in the patients. Fourth, pathways that have low CNI and high CNAs highlight putative passenger CN events. We found that of the 85 putative CN-driven pathways at diagnosis (Fig. 3 and Supplementary Table 3) 18 pathways were significantly enriched in poor responders, 36 were enriched in good responders, and 31 were common to both groups.

To capture the systematic changes in CNI over the treatment phase, we implemented the stacked nonlinear version of our model (Methods). Interestingly, the CNI changes over treatment (177 treatment-naïve *vs.* 56 chemotherapy treated samples) was significant with very low CNI for only one pathway (Beta2 integrin cell surface interactions; *R* = 0.1, *p* < 2.2e-16). This suggests that the mechanisms behind functional CNAs remain stable during chemotherapy, and that they can be identified already from the treatment-naïve samples.

The CNI and CNA landscape for all DECIDER samples, including all treatment phases and treatment responses, are displayed across the four clusters in Supplementary Fig. 4 providing an overview of the landscape for the entire cohort. As the CNI values do not significantly change during chemotherapy, the pathways with high CNI contribution are likely to have prognostic power.

### CNI quantification reveals CN-driven pathways with prognostic significance

**Fig. 4:**
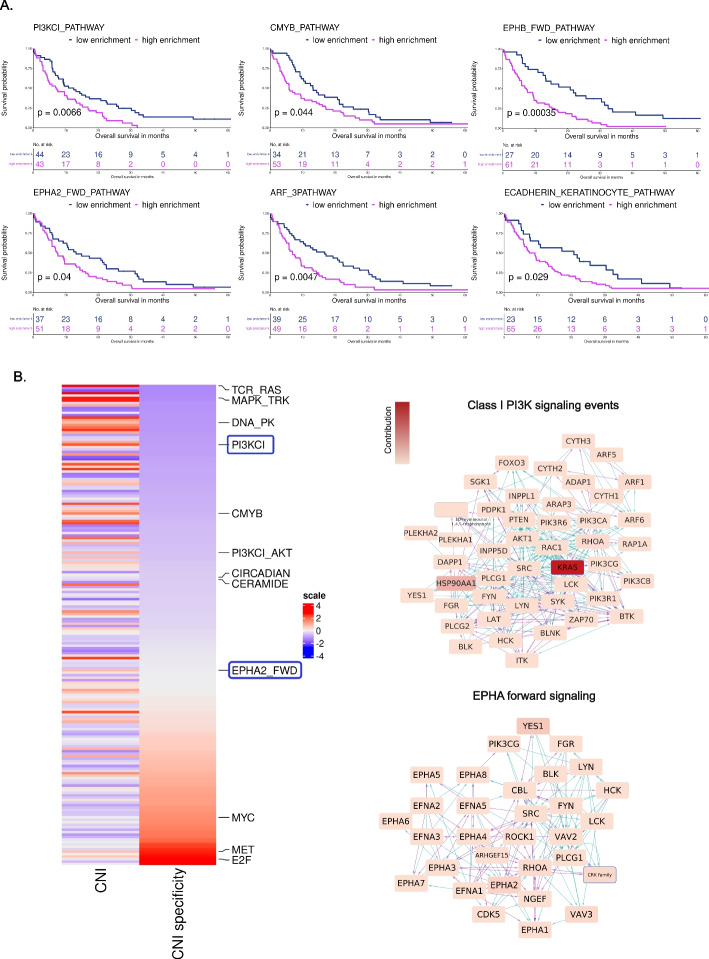
Pathways with ssGSEA scores associated to the patients’ survival and their specificity **A**. Kaplan–Meier survival curves of six pathways which were found to be significantly associated with unfavorable PFI in a two-group analysis based on an optimized ssGSEA threshold using the max.stat method in DECIDER cohort. **B**. The quantified CNI specificity values across the 196 PID pathways along with the CNI values. Pathways are ordered based on their CNI gene specificity. Two examples of diffuse and specific pathways are shown, along with the genes that most significantly contribute to their CNI

To test whether pathways with high CNI contribution are more prognostic than the less CNA regulated, we used pathway level ssGSEA scores [33]. We calculated the pathway scores for the 177 treatment-naïve samples in the DECIDER cohort and calculated their association to platinum-free interval (PFI). Among the 18 pathways enriched in poor-responding patients, six exhibited statistical significance (Fig. 4A). Supplementary Figs. 5 and 6 depict the gene expression profiles and CNAs for these pathways. The top contributing genes to CNI in these six pathways, are detailed in Supplementary Table 4, showing each gene’s contribution and their genomic localization. Additionally, Supplementary Fig. 7 represents the gene expression correlation among the top contributing genes of candidate pathways in diagnostic samples.

To explore the associations between the three other pathway categories and platinum-free interval (PFI), we also investigated non-CN-driven (*n* = 28), passenger CN (*n* = 21), and conserved CN (*n* = 62) pathways. We found that no pathways in the non-CN-driven and passenger CN categories were significantly associated with PFI. In the conserved CN category, only three pathways (Notch-mediated HES/HEY network, Regulation of nuclear SMAD2/3 signaling and FAS (CD95) signaling pathway) showed a significant association with PFI.

### Pathway level CNI analysis suggests *KRAS* as a treatment resistance driver

We observed that many pathways have only a single or a few genes with high CNI values, whereas others have a more uniform CNI distribution. This suggests that pathways whose activity is driven by a single or few genes provide opportunities for effective interventions. Thus, we quantified the contributions of the genes in the 196 PID pathways. We first quantified the contribution of the involved genes to the aggregate pathway CNI. Then, from the relative explained variance by each involved gene, we calculated the effective number of pathway activity-contributing genes for each individual pathway (Methods). Some pathways displayed specific CNI patterns implying that aberrations are caused by few genes, whereas others showed more dispersed CNI patterns indicating that multiple genes within these pathways showed similar dysregulation in terms of CNAs (Fig. 4B).

Interestingly, three out of six pathways associated with poor treatment response (Class I PI3K signaling events, C-MYB transcription factor network, EPHB forward signaling) had *KRAS* as the single dominant gene exhibiting contribution of 36% to 46% (Supplementary Table 5) with the individual contributions by the other genes being < 8%. Indeed, Class I PI3K signaling events has *KRAS* as the major contributor with a 46% contribution, followed by *HSP90AA1* with 8%. Similarly, the C-MYB transcription factor network has *KRAS* as the primary contributor with 41% contribution, followed by HSPA8 with 7%. Lastly, EPHB forward signaling shows *KRAS* as the principal contributor with a 36% contribution, followed by *TF* with a 3% contribution.

In the DECIDER cohort, the size of the subset of patients with HGSC and functional wild-type KRAS amplification (wt*KRAS*^*amp*^) is ~ 13%. Of note, none of the patients in the DECIDER has *KRAS* mutations. We then tested whether *KRAS* amplifications have independent survival association and analyzed *KRAS* gene expression and CNAs separately for the 88 treatment-naïve patients. The *KRAS* gene expression was significantly associated with PFI (*p* = 0.012) and OS (*p* = 0.0028) in the DECIDER cohort, as shown in Supplementary Fig. 8. The *KRAS* gene expression was also associated with unfavorable OS in TCGA OV cohort (*p* = 0.047). For the CNA survival association, we used the functional CNA threshold of seven copies, resulting in 12 patients with wt*KRAS*^*amp*^, and 75 patients with unamplified wt*KRAS.* We observed a similar, albeit not significant, survival association trend as with *KRAS* gene expression (PFI: *p* = 0.46; OS: *p* = 0.08). Out of the 12 patients with wt*KRAS*^*amp*^, nine had high gene expression values that were associated with poor survival, which supports the utility of CNI values in finding functionally relevant CNAs. 

### *KRAS* decreases platinum sensitivity in ovarian cancer cells

**Fig. 5:**
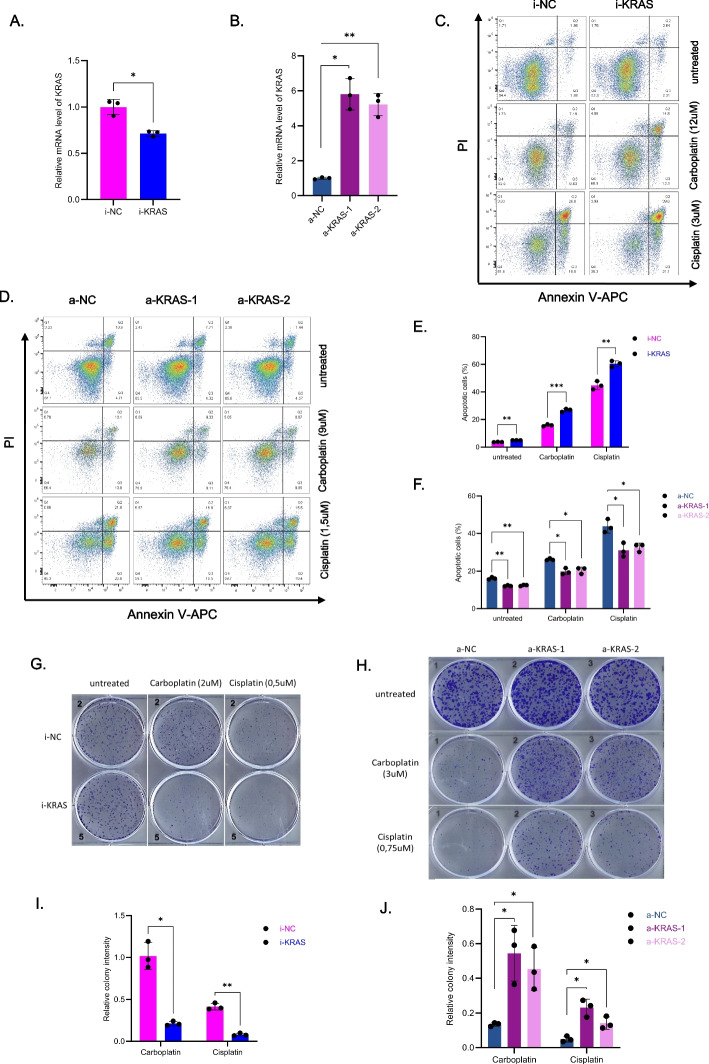
*KRAS *promotes platinum resistance in ovarian cancer cells **A**,** B**. qRT-PCR was performed to determine *KRAS* mRNA levels in KURAMOCHI-i-NC (negative control sgRNA for the inhibition system), KURAMOCHI-i-KRAS (*KRAS* inhibition sgRNA), CAOV3-a-NC (negative control sgRNA for the activation system), CAOV3-a-KRAS-1 (*KRAS* activation sgRNA #1) and CAOV3-a-KRAS-2 (*KRAS* activation sgRNA #2). RPS13 was used as a housekeeping gene. **C**,** D**. Flow cytometry assay was used to detect apoptotic cells in KURAMOCHI (**C**) after 72 h treatment with carboplsatin (12 μM) and cisplatin (3 μM) or CAOV3 (**D**) with carboplatin (9 μM) and cisplatin (1.5 μM). **E**,** F**. Quantification of percentage of apoptotic cells from (**C**) and (**D**). **G**,** H**. Colony formation assay was performed to verify the colony formation ability of KURAMOCHI (**G**) and CAOV3 (**H**) after 7 days of presence of the indicated concentration of platinum plus another 7 days after removing platinum. **I**,** J** Quantification of the colony intensity from (**G**) and (**H**) after normalization by untreated condition. (Student’s t test were used; NS p > 0.05, * *p* < 0.05, ** *p* < 0.01, *** *p* < 0.001; replicates: *n* = 3)

To experimentally validate our findings regarding the role of *KRAS* in treatment resistance, we examined how modulation of *KRAS* levels affects platinum response in two HGSC cell lines, KURAMOCHI, which has a *KRAS* amplification and CAOV3 that is *KRAS* copy-number neutral [46]. For KURAMOCHI, we generated *KRAS* downregulated cells via CRISPR inhibition [47] and for CAOV3, we upregulated *KRAS* by CRISPR activation [37]. qRT-PCR was performed to confirm the down- and upregulation efficiency of *KRAS* in these cells, with around 30% reduction and 4.5x induction (Fig. 5A, B, respectively).

We first assessed effects of increased levels of wt*KRAS* to cell viability and short-term platinum response. The knockdown of *KRAS* significantly increased the proportion of apoptotic cells in both untreated control cells and after 3-day carboplatin (12 μM) or cisplatin (3 μM) treatment in the KURAMOCHI cell line (Fig. 5C, E). However, *KRAS* inhibition increased the level of apoptotic cells significantly only in carboplatin treatment when normalized to untreated control (Supplementary Fig. 9A). In CAOV3, up-regulation of *KRAS* reduced the percentage of apoptotic cells in both untreated and carboplatin (9 μM) or cisplatin (1.5 μM) treated cells to a similar extent (Fig. 5D, F, Supplementary Fig. 9B). These results suggest that increased *KRAS* levels are associated with improved viability in both intrinsically *KRAS* amplified and CN neutral contexts but provide no or minimal additional benefit in short-term platinum treatment.

To analyze the effect of *KRAS* perturbation in a prolonged platinum treatment by using lower, more clinically relevant concentrations of platinum drugs, we performed colony formation assays with one week of treatment and one week of recovery. Here, the effects of *KRAS* on platinum resistance were more pronounced and significant in both cell line pairs. KURAMOCHI cells were considerably more sensitive to platinum after *KRAS* down-regulation (Fig. 5G, I, Supplementary Fig. 9C), while *KRAS* up-regulation made CAOV3 cells highly resistant to both carboplatin and cisplatin (Fig. 5H, J, Supplementary Fig. 9D). These results indicate that increased *KRAS* levels improve viability, and drive chemoresistance in functional models that mimic the effects of wild-type *KRAS* amplification.

## Conclusion

While copy-number alterations (CNAs) are prominent in many cancers [2] different cancer types exhibit varying degrees of CNAs, allowing classification to mutation or CN dominant cancers [6]. Herein, we hypothesized that systematic and robust discovery of genome regions that have direct and strong impact on the mRNA levels of the genes in these regions is integral to understand cancer progression and treatment resistance. To identify such genes and pathways in an unbiased and systematic fashion, we developed an approach that quantifies the degree to which CNAs regulate expression levels. We applied this approach to 14 cancer types and found significant variations in the copy-number impact (CNI) on expression levels. Our results show that HGSC is characterized by high levels of CNAs and a high CNI, followed by lung squamous cell carcinoma (LUSC) as expected [6].

Our analysis revealed that dividing pathways into four clusters based on their CNA level and the CNI characterized by our model distinguishes putative driver and passenger CNA changes. Interestingly, some pathways in the copy-number driver cluster with high CNA level and high CNI were prognostic at diagnosis. These results highlight that the analysis of CNAs, while taking their impact on expression levels into account, enables us to understand the role of CNAs and identify clinically relevant alterations in genomically unstable cancers such as HGSC.

The CNI analysis identified several pathways that are likely driven by genes whose expression levels are regulated by CNAs. Interestingly, three out of the six poor response associated pathways were dominated by the *KRAS* gene. Furthermore, EPHA2 forward signaling and Arf1 pathway, both showing a more diffuse pattern of CNI, are also closely linked to *KRAS* [48, 49]. High expression of the *KRAS* gene was significantly associated with short PFI. While activating mutations in *KRAS* have been studied extensively in low-grade serous [50] and other cancers, in HGSC [51] the activating mutations are almost non-existent and the role of wt*KRAS* has been less explored [52]. Our results indicate that the wt*KRAS*^*amp*^ plays an important role in treatment response for a subset of patients with HGSC. In the DECIDER cohort, the size of this subset of patients with HGSC and functional wt*KRAS*^*amp*^ is ~ 13% and none of DECIDER patients had *KRAS* mutations. 

To functionally test the effect of *KRAS* amplification on platinum response, we mimicked the modulation of *KRAS* CNAs by CRISPR interference in a cell line context. Here, *KRAS* induction drove chemoresistance in intrinsically copy-number-neutral cells, and repression rendered *KRAS* amplified cells more sensitive to both cisplatin and carboplatin. Previously, the effects of wt*KRAS* amplification have been studied in the context of tyrosine kinase inhibitor resistance [53, 54] whereas the effects to platinum chemotherapy response have been unexplored. Our findings demonstrate that *KRAS* plays a functional role in platinum resistance, and suggest wt*KRAS*^*amp*^-induced chemoresistance as a putative cause for the shortened PFI in patients with high *KRAS* expression.

The current success in developing effective inhibitors for wt*KRAS*^*amp*^ tumors [55] underscores the substantial translational value of the role for wt*KRAS*^*amp*^ in HGSC. Identification of a specific subgroup of HGSC patients who might exhibit resistance to existing standard-of-care treatments but could greatly benefit from therapies specifically designed to target wt*KRAS*^*amp*^ tumor is of high importance to improve treatment outcomes.

In addition to the importance of wt*KRAS*^*amp*^, our pathway level analysis identified three pathways that were associated with poor response at diagnosis. Interestingly, these pathways involve multiple genes that collectively contribute to pathway dysregulation manifesting as similar malignant phenotypes. This highlights the strength of our method in identifying prognostic mechanisms at the pathway level with convergent dysregulation due to distinct CNAs.

The main limitation of this study is that in addition to CNAs, there are other processes that affect gene regulation, such as point mutations [56], epigenetic aberrations [57], and phenotypic diversity [58] which we have not considered in this study. However, the model can be modified to be applicable to other data layers and this is one of our future directions. These additional data layers could potentially offer insights for conserved and passenger CNA clusters. Furthermore, our pathway analyses were based on gene expression data, while pathways function at the protein level, highlighting the need for further investigation of the pathways identified.

Taken together, we developed an approach that enables identifying CNAs that have a strong and measurable impact on gene expression values. This allows pinpointing the genes and pathways that are likely to be more important in cancer progression and therapy resistance, as abundant CNAs alone can lead to high levels of noise and manifests as passenger findings. Our approach also provides means to identify the most important alterations contributing to pathway dysregulation, which can be used to reveal contexts in which a gene contributes to therapy resistance. Importantly, our results demonstrate the importance of wt*KRAS*^*amp*^ in HGSC driving the chemotherapy resistance.

### Supplementary Information

**Additional file 1: Supplementary results.** Copy number impact (CNI) on gene expression. **Supplementary Figure 1.** Examples of copy-number gene expression models. **Supplementary Figure 2.** CNI over all genes across different cancers, **Supplementary Figure 3.** Changes in CNI over the HGSC response groups, **Supplementary Figure 4.** The landscape of CNA versus CNI in the DECIDER HGSC cohort, **Supplementary Figure 5.** Pathway enrichment scores between the HGSC response groups of the potential CN driven pathways, **Supplementary Figure 6.** CNAs between the HGSC response groups of the potential CN driven pathways, **Supplementary Figure 7.** Gene expression correlation among top contributing genes to CNI in six pathways associated to survival., **Supplementary Figure 8.** KRAS CNA level and gene expression association with patient surviva, **Supplementary Figure 9.** Quantification of apoptotic cells percentage and colony intensities, **Supplementary Table 1.** CNA versus CNI across four driver gene sets in different cancers, **Supplementary Table 2.** CNA functional transition point and its range across whole genome, **Supplementary Table 3.** PID pathways and association to response groups, **Supplementary Table 4.** Characteristics of top genes in six survival associated pathways, **Supplementary Table 5.** Specificity of functional CNAs in the PID pathways, **Supplementary Table 6.** DECIDER HGSC cohort sample information, **Supplementary Table 7.** DECIDER HGSC cohort sample information, **Supplementary Table 8.** Primer/sgRNA sequences

## Data Availability

All raw DNA sequencing data is submitted to the European Genome-phenome Archive (EGA) and will be publicly available under study accession number EGAS00001006775. Raw bulk RNA sequencing data are deposited in the EGA and are publicly available (EGAS00001004714).
