# Supplementary material for: Genome-wide quantification of copy-number aberration impact on gene expression in ovarian high-grade serous carcinoma
Source: BMC Cancer. 2024 Feb 5;24:173. doi: 10.1186/s12885-024-11895-6 (PMC10840274; doi:10.1186/s12885-024-11895-6)
Supplement: Supplementary file 1 — Additional file 1: Supplementary results. Copy number impact (CNI) on gene expression. Supplementary Figure 1. Examples of copy-number gene expression models. Supplementary Figure 2. CNI over all genes across different cancers, Supplementary Figure 3. Changes in CNI over the HGSC response groups, Supplementary Figure 4. The landscape of CNA versus CNI in the DECIDER HGSC cohort, Supplementary Figure 5. Pathway enrichment scores between the HGSC response groups of the potential CN driven pathways, Supplementary Figure 6. CNAs between the HGSC response groups of the potential CN driven pathways, Supplementary Figure 7. Gene expression correlation among top contributing genes to CNI in six pathways associated to survival., Supplementary Figure 8. KRAS CNA level and gene expression association with patient surviva, Supplementary Figure 9. Quantification of apoptotic cells percentage and colony intensities, Supplementary Table 1. CNA versus CNI across four driver gene sets in different cancers, Supplementary Table 2. CNA functional transition point and its range across whole genome, Supplementary Table 3. PID pathways and association to response groups, Supplementary Table 4. Characteristics of top genes in six survival associated pathways, Supplementary Table 5. Specificity of functional CNAs in the PID pathways, Supplementary Table 6. DECIDER HGSC cohort sample information, Supplementary Table 7. DECIDER HGSC cohort sample information, Supplementary Table 8. Primer/sgRNA sequences [file 12885_2024_11895_MOESM1_ESM.zip › Supplemetry Material/Supplementary_Fig4.pdf]

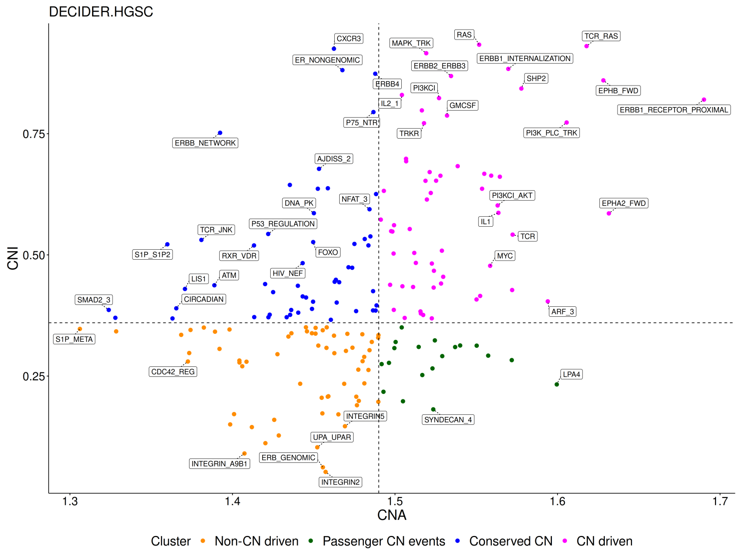
DECIDER.HGSC
CNI
CNA
0.75
0.50
0.25
1.3
1.4
1.5
1.6
1.7
CXCR3
ER_NONGENOMIC
MAPK_TRK
RAS
TCR_RAS
ERBB2_ERBB3
ERBB1_INTERNALIZATION
ERBB4
PI3KCI
SHP2
EPHB_FWD
IL2_1
GMCSF
ERBB1_RECEPTOR_PROXIMAL
P75_NTR
TRKR
PI3K_PLC_TRK
ERBB_NETWORK
AJDISS_2
NFAT_3
DNA_PK
PI3KCI_AKT
EPHA2_FWD
IL1
P53_REGULATION
TCR_JNK
TCR
S1P_S1P2
RXR_VDR
FOXO
MYC
HIV_NEF
LIS1
ATM
CIRCADIAN
SMAD2_3
ARF_3
S1P_META
CDC42_REG
LPA4
INTEGRIN5
SYNDECAN_4
UPA_UPAR
ERB_GENOMIC
INTEGRIN_A9B1
INTEGRIN2
Cluster
Non-CN driven
Passenger CN events
Conserved CN
CN driven
