# Supplementary material for: Genome-wide quantification of copy-number aberration impact on gene expression in ovarian high-grade serous carcinoma
Source: BMC Cancer. 2024 Feb 5;24:173. doi: 10.1186/s12885-024-11895-6 (PMC10840274; doi:10.1186/s12885-024-11895-6)
Supplement: Supplementary file 1 — Additional file 1: Supplementary results. Copy number impact (CNI) on gene expression. Supplementary Figure 1. Examples of copy-number gene expression models. Supplementary Figure 2. CNI over all genes across different cancers, Supplementary Figure 3. Changes in CNI over the HGSC response groups, Supplementary Figure 4. The landscape of CNA versus CNI in the DECIDER HGSC cohort, Supplementary Figure 5. Pathway enrichment scores between the HGSC response groups of the potential CN driven pathways, Supplementary Figure 6. CNAs between the HGSC response groups of the potential CN driven pathways, Supplementary Figure 7. Gene expression correlation among top contributing genes to CNI in six pathways associated to survival., Supplementary Figure 8. KRAS CNA level and gene expression association with patient surviva, Supplementary Figure 9. Quantification of apoptotic cells percentage and colony intensities, Supplementary Table 1. CNA versus CNI across four driver gene sets in different cancers, Supplementary Table 2. CNA functional transition point and its range across whole genome, Supplementary Table 3. PID pathways and association to response groups, Supplementary Table 4. Characteristics of top genes in six survival associated pathways, Supplementary Table 5. Specificity of functional CNAs in the PID pathways, Supplementary Table 6. DECIDER HGSC cohort sample information, Supplementary Table 7. DECIDER HGSC cohort sample information, Supplementary Table 8. Primer/sgRNA sequences [file 12885_2024_11895_MOESM1_ESM.zip › Supplemetry Material/Supplementary_Fig7.pdf]

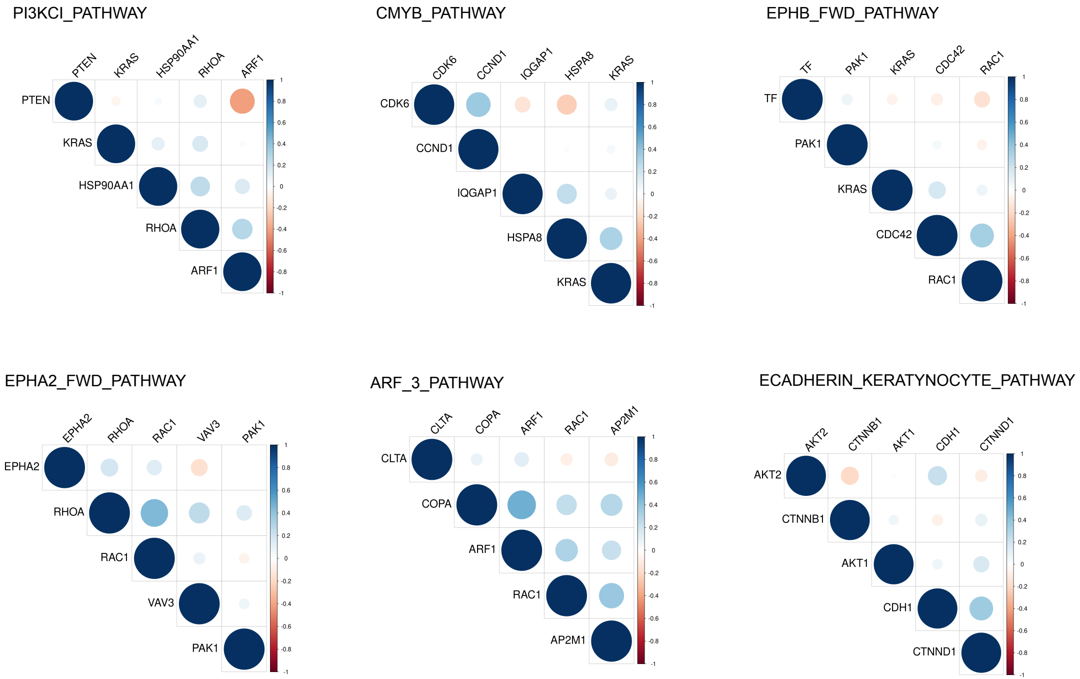
PI3KCI_PATHWAY
PTEN
KRAS
HSP90AA1
RHOA
ARF1
CMYB_PATHWAY
CDK6
CCND1
IQGAP1
HSPA8
KRAS
EPHB_FWD_PATHWAY
TF
PAK1
KRAS
CDC42
RAC1
EPHA2_FWD_PATHWAY
EPHA2
RHOA
RAC1
VAV3
PAK1
ARF_3_PATHWAY
CLTA
COPA
ARF1
RAC1
AP2M1
ECADHERIN_KERATYNOCYTE_PATHWAY
AKT2
CTNNB1
AKT1
CDH1
CTNND1
1
0.8
0.6
0.4
0.2
0
-0.2
-0.4
-0.6
-0.8
-1
