# Supplementary material for: Genome-wide quantification of copy-number aberration impact on gene expression in ovarian high-grade serous carcinoma
Source: BMC Cancer. 2024 Feb 5;24:173. doi: 10.1186/s12885-024-11895-6 (PMC10840274; doi:10.1186/s12885-024-11895-6)
Supplement: Supplementary file 1 — Additional file 1: Supplementary results. Copy number impact (CNI) on gene expression. Supplementary Figure 1. Examples of copy-number gene expression models. Supplementary Figure 2. CNI over all genes across different cancers, Supplementary Figure 3. Changes in CNI over the HGSC response groups, Supplementary Figure 4. The landscape of CNA versus CNI in the DECIDER HGSC cohort, Supplementary Figure 5. Pathway enrichment scores between the HGSC response groups of the potential CN driven pathways, Supplementary Figure 6. CNAs between the HGSC response groups of the potential CN driven pathways, Supplementary Figure 7. Gene expression correlation among top contributing genes to CNI in six pathways associated to survival., Supplementary Figure 8. KRAS CNA level and gene expression association with patient surviva, Supplementary Figure 9. Quantification of apoptotic cells percentage and colony intensities, Supplementary Table 1. CNA versus CNI across four driver gene sets in different cancers, Supplementary Table 2. CNA functional transition point and its range across whole genome, Supplementary Table 3. PID pathways and association to response groups, Supplementary Table 4. Characteristics of top genes in six survival associated pathways, Supplementary Table 5. Specificity of functional CNAs in the PID pathways, Supplementary Table 6. DECIDER HGSC cohort sample information, Supplementary Table 7. DECIDER HGSC cohort sample information, Supplementary Table 8. Primer/sgRNA sequences [file 12885_2024_11895_MOESM1_ESM.zip › Supplemetry Material/Supplementary material.pdf]

Supplementary material

Genome-wide Quantification of Copy-Number Aberration Impact on Gene Expression in Ovarian High-Grade Serous Carcinoma

Sanaz Jamalzadeh[1], Jun Dai[1], Kari Lavikka[1], Yilin Li[1], Jing Jiang[1], Kaisa Huhtinen[1,2], Anni Virtanen[3], Jaana Oikkonen[1], Sakari Hietanen[4], Johanna Hynninen[4], Anna Vähärautio[1,5], Antti Häkkinen[1,6,†], Sampsa Hautaniemi[1,†]

Supplementary results

Copy number impact (CNI) on gene expression

Linear model of CNI. We used a linear Poisson model to study whether the copy-number alterations (CNAs) exert non-linear changes in the gene expression. A linear model captured the variation for some genes quite well (*CCNE1*), while for the majority of the genes the nonlinear model outperformed the linear model significantly, as e.g. for *ERBB2* which has a linear correlation of 65% and a nonlinear correlation of 92%. Overall, the non-linear model resulted in capturing significantly more gene expression variation compared to the linear one, as evaluated by the likelihood ratio test ($p < 0.001$), suggesting the CNA effects on gene expression are globally highly nonlinear (cf. Supplementary Figure 1). *RB1*, *ERBB2,* and *CCNE1* highlight genes where the model indicates globally a high CNI and high functional impact on gene expression, while *MET*, *MUC4,* and *FOXA1* represent genes with low CNI and consequently the abundance of non-functional CNAs. Additionally, a high CNI can stem from specific deletions (*RB1*), high-level amplifications (e.g. *ERBB2*), or vary broadly with the exact CNA level (e.g. *CCNE1*).

CNI of known copy-number and mutational drivers in the DECIDER cohort. We selected some of the previously established CN drivers from a TCGA CN driver gene set [1] (cn.dbv3) consisting of 182 genes, and investigated the quantified CNI among those drivers. For instance, *MYCN* has a CNI of 54% over its gene expression, *NRAS* and *KRAS* CNAs control their gene expression with a CNI of 43% and 97%, respectively. *ERBB2* controls its expression with an impact of 92%, *CCNE1* with 74%, *MMP7* with 83%, and *PAK1* has a CNI of 96% over its gene expression (all significant with $p < 0.001$). Overall, in the cn.dbv3 gene set, the CNI values are significantly enriched compared to the whole genome ($p < 2.2e{-16}$ in a t-test between the cn.dbv3 gene set and 100 randomly selected

gene sets). The results show that the CNI quantification identified the functional impact of the previously known drivers from the TCGA cohort.

CNI of known copy-number and mutational drivers across cancers. We also analyzed the TCGA pan-cancer gene set consisting of 182 copy-number driver genes (cn.dbv3) [1] for the other cohorts, along with another gene set consisting of 2,166 mutation driver genes identified in TCGA (mut.dbv3) [1], a gene set of 318 genes studied in pan-cancer patterns of somatic copy-number alteration (cn.amp.ann) [2], and 34 differentially expressed genes correlated with copy-number variation (cn.deg) [3]. As expected, across the cancer types, the putative copy-number drivers are located in the region of high CNA and high CNI (see Supplementary Table 1), while the putative mutational drivers are located in the low CNA and low CNI region.

Gene expression and CNAs of the response group specific pathways

The box-plot representation of gene expression profiles over the response groups are shown in Supplementary Figure 5 for the pathways enriched in poor responder specific CNAs. We also investigated whether the CNAs of poor responder specific pathways could be used to prognosticate the patients. We used the CNAs for each gene and quantified each pathway's dominant CNA status as deletion, loss, normal, gain, or amplification. As shown in Supplementary Figure 6, we found more CNA gains in poor responders, whereas more normal states are consistently found in the good responders, regardless of pathway specificity of the functional CNAs. This suggests that CNA losses and gains play distinct functional roles in expression, reinforcing the mechanistic differences in CNA function between the two response groups.

References

1. Liu, S. H. *et al.* DriverDBv3: a multi-omics database for cancer driver gene research. *Nucleic Acids Res.* **48**, (2020).

2. Zack, T. I. *et al.* Pan-cancer patterns of somatic copy number alteration. *Nat. Genet.* **45**, (2013).

3. Shao, X. *et al.* Copy number variation is highly correlated with differential gene expression: a pan-cancer study. *BMC Med. Genet.* **20**, 1–14 (2019).

Supplementary Figures

Supplementary Figure 1. Examples of copy-number gene expression models

Gene expression as a function of the corresponding absolute CNAs, with the data versus expression models for six genes including *RB1*, *ERBB2*, *CCNE1*, *FOXA1*, *MET,* and *MUC4*. Both the optimal non-linear and linear models are shown, along with the average expression.

Supplementary Figure 2. CNI over all genes across different cancers

Gene level quantification of the CNI in the DECIDER HGSC and TCGA OV as CN driven cancers compared with the TCGA SKCM and TCGA GBM, exemplifying less CN driven cancers. The genes with captured significant and non-significant CNI are highlighted with different colors.

Supplementary Figure 3. Changes in CNI over the HGSC response groups

CNI changes over response groups in diagnosis samples, as captured by the generalized correlation coefficients for each gene. The genes with significant and non-significant CNI change are highlighted with different colors.

Supplementary Figure 4. The landscape of CNA versus CNI in the DECIDER HGSC cohort

Clustering of 196 PID pathways on the combined landscape of CNA versus CNI in the DECIDER HGSC cohort. The magenta points represent the putative CN driven pathways, blue the conserved CN, orange the non-CN driven pathways, and the green passenger CN events.

Supplementary Figure 5. Pathway enrichment scores between the HGSC response groups of the potential CN driven pathways

The box-plot representation of the ssGSEA scores for each of the response groups for the 18 poor responder specific pathways.

Supplementary Figure 6. CNAs between the HGSC response groups of the potential CN driven pathways

Pathway level CNA status as deletion, loss, normal, gain, or amplification in pathways enriched with functional CNAs in the poor responder patients.

Supplementary Figure 7. Gene expression correlation among top contributing genes to CNI in six pathways associated to survival.

Gene expression correlation among top contributing genes to pathways CNI for six pathways associated to survival. The gene expression profiles are coming from diagnosis samples in DECIDER cohort.

Supplementary Figure 8. *KRAS* CNA level and gene expression association with patient survival

A. Association with the *KRAS* gene expression level and overall survival or platinum-free interval in 88 treatment-naïve samples from 88 patients in the DECIDER cohort. **B.** *KRAS* amplification status association with the overall survival or platinum-free interval for 89 treatment-naïve samples from 88 patients in the DECIDER cohort.

Supplementary Figure 9. Quantification of apoptotic cells percentage and colony intensities

A. Quantification of the apoptotic cells percentage from KURAMOCHI-i-NC (negative control sgRNA for the inhibition system), KURAMOCHI-i-KRAS (KRAS inhibition sgRNA) after carboplatin or cisplatin treatment after normalization by the untreated condition. **B.** Quantification of the apoptotic cells percentage from CAOV3-a-NC (negative control sgRNA for the activation system), CAOV3-a-KRAS-1 (KRAS activation sgRNA #1) and CAOV3-a-KRAS-2 (KRAS activation sgRNA #2) after carboplatin or cisplatin treatment after normalization by the untreated condition. **C, D.** Quantification of the colony intensity of KURAMOCHI (C) and CAOV3 (D).
